# Big Data-Planetary Health approach for evaluating the Brazilian Dengue Control Program

**DOI:** 10.11606/s1518-8787.2024058005491

**Published:** 2024-04-12

**Authors:** Fernando Xavier, Gerson Laurindo Barbosa, Cristiano Corrêa de Azevedo Marques, Antonio Mauro Saraiva

**Affiliations:** I Universidade de São Paulo Programa de Pós-Graduação em Engenharia Elétrica São Paulo SP Brasil Universidade de São Paulo. Programa de Pós-Graduação em Engenharia Elétrica. São Paulo, SP, Brasil; II Secretaria de Estado da Saúde de São Paulo Instituto Pasteur São Paulo SP Brasil Secretaria de Estado da Saúde de São Paulo Instituto Pasteur. Technical Area of Diseases Linked to Vectors and Intermediate Hosts. São Paulo, SP, Brasil; III Universidade de São Paulo Escola Politécnica Departamento de Engenharia de Computação e Sistemas Digitais São Paulo SP Brasil Universidade de São Paulo. Escola Politécnica. Departamento de Engenharia de Computação e Sistemas Digitais. São Paulo, SP, Brasil

**Keywords:** Epidemiology, Dengue, Outcome and Process Assessment, Health Care, Data Science

## Abstract

**OBJECTIVE:**

This study aims to integrate the concepts of planetary health and big data into the Donabedian model to evaluate the Brazilian dengue control program in the state of São Paulo.

**METHODS:**

Data science methods were used to integrate and analyze dengue-related data, adding context to the structure and outcome components of the Donabedian model. This data, considering the period from 2010 to 2019, was collected from sources such as Department of Informatics of the Unified Health System (DATASUS), the Brazilian Institute of Geography and Statistics (IBGE), WorldClim, and MapBiomas. These data were integrated into a Data Warehouse. K-means algorithm was used to identify groups with similar contexts. Then, statistical analyses and spatial visualizations of the groups were performed, considering socioeconomic and demographic variables, soil, health structure, and dengue cases.

**OUTCOMES:**

Using climate variables, the K-means algorithm identified four groups of municipalities with similar characteristics. The comparison of their indicators revealed certain patterns in the municipalities with the worst performance in terms of dengue case outcomes. Although presenting better economic conditions, these municipalities held a lower average number of community healthcare agents and basic health units per inhabitant. Thus, economic conditions did not reflect better health structure among the three studied indicators. Another characteristic of these municipalities is urbanization. The worst performing municipalities presented a higher rate of urban population and human activity related to urbanization.

**CONCLUSIONS:**

This methodology identified important deficiencies in the implementation of the dengue control program in the state of São Paulo. The integration of several databases and the use of Data Science methods allowed the evaluation of the program on a large scale, considering the context in which activities are conducted. These data can be used by the public administration to plan actions and invest according to the deficiencies of each location.

## INTRODUCTION

The number of dengue cases and deaths has been increasing every year, surpassing historical records. According to the Brazillian Ministry of Health Bulletin for the 52nd epidemiological week of 2022^1^, 992 confirmed dengue deaths were reported, a higher number than in 2015 (986), when the highest number had been recorded so far^2^. According to the same bulletin, the state of São Paulo presented the highest number of deaths that year (278), about 28% of the total registered in the country.

In Brazil, vector control of *Aedes* began in 1976 with sporadic actions, and since 2002, with the creation of the *Programa Nacional de Controle da Dengue* (PNCD – Brazilian Dengue Control Program), routine activities have been developed to combat the vector. Some of these activities, such as larval sampling research in households, visits to strategic points, home visits aimed at guiding the residents and eliminating immature forms of the vector by implementing transmission blocking agents when necessary, among others, are routinely developed in the state of São Paulo, following national guidelines.

The dengue-transmitting vector (the *Aedes aegypti* mosquito) finds favorable conditions for its development in places with higher temperatures and highly urbanized environments. According to projections, these places are likely to increase in the coming years^3^. Given the strong influence of changes in the environment for diseases such as dengue^4^, climate change may contribute to the increased proliferation of vectors. Urbanization without adequate planning represents another threat to several infectious diseases^5^, and, with the worsening of living conditions, the migratory flow may increase even more. Over the past decade, an average of 24 million people per year have been displaced from their homes due to climate-related events^6^.

Although urbanization can be seen as a reflection of economic development, it does not always occur adequately^7^. The destruction of ecosystems and the disorderly occupation of spaces cause several impacts on the living conditions of the population^7^. While some indicators have improved, such as life expectancy, others have worsened, such as the frequency of disease outbreaks^8^.

This complex relationship between human health and environmental conditions is the subject of a study in the area called Planetary Health^9^, in which research seeks to assess the impact of the environment on health. Health conditions, which are often a reflection of human activity, are related to this context. Dengue cases are, among other factors, related to climatic conditions^10^ that, in turn, can be related to human activity^9^.

Thus, the assessment of health conditions may be related to the context in which the activities are performed. The Donabedian model^11^ considers that health outcomes depend on processes and structure. In this model, evaluations can include analyses of three components that are related to each other: structure, process, and outcome.

Evaluating the indicators of the structure component allows us to analyze the situation of the health service structure in the area: the number of professionals, care units, investments, among others. Process indicators, in turn, evaluate whether care processes are being performed appropriately, for example. These two components can be evaluated in conjunction with outcome indicators, allowing for an integrated evaluation of the conditions under which health outcomes are produced.

However, the conditions and actions required for health outcomes may also depend on the context in which they are inserted^12^. Thus, health evaluations should also consider context-related variables.

Considering the complex relationship between health determinants discussed in the Planetary Health research area, health evaluation methods, such as the Donabedian model, could use data from different dimensions, such as the economy, living conditions, and urban development. However, the increasing complexity of the health evaluation process can hinder this process due to cost and time constraints, especially in the context of Big Data, in which multiple sources of data are generated at ever-increasing speeds.

In this sense, by adopting Data Science techniques, such as the application of machine learning algorithms, and statistical analysis, this study aims to aggregate context in the evaluation of the Brazilian Dengue Control Program based on the Donabedian model. Given the diversity of variables related to dengue, the use of Data Science can bring great benefits to the subject of health evaluation, such as allowing evaluation processes to be performed on a large scale. In this study, data from several variables of the 645 municipalities in the state of São Paulo, from 2010 to 2019, were analyzed.

## METHODS

Research based on Data Science considers, in addition to computational and statistical techniques, the inclusion of domain experts^13^. This interaction adds the vision/human insight of a field to the research, including identifying the necessary data sources, defining the problem, and evaluating the outcomes. Thus, this study is divided into six stages, described below, from the definition of the evaluation model to the analysis of the outcomes.

### Definition of the evaluation model

The main indicators for the evaluation of the Brazilian Dengue Combat Program were identified. This process relied on existing literature to add formal evidence for the use of the indicators^14^, as well as validation by domain experts in the matrix shown in Chart 1. For this research, indicators related to the structure and outcome components of the Donabedian model were used, in addition to those related to the context.

**Chart:** Evaluation matrix used, considering Context, Structure, and Outcome.

Indicator	Description	Type	Source
Context			
Tmax	Maximum mean annual temperature	Climate	WorldClim
Tmin	Minimum mean annual temperature	Climate	WorldClim
Prec	Annual precipitation volume	Climate	WorldClim
GDP	Gross domestic product *per capita*	Socioeconomic	IBGE
Gini	Gini Index	Socioeconomic	DATASUS
HDI	Human development index	Socioeconomic	IPEA
Infant mortality	Infant mortality rate per 1000 live births	Health	DATASUS
Water	Percentage of population with access to water services	Sanitation	SNIS
Sewage	Percentage of population with access to sewage services	Sanitation	SNIS
Urban population	Percentage of urban population	Demographic	SNIS
NAgri	Percentage of total area modified for agricultural activities	Soil use	MAPBIOMAS.
NPast	Percentage of total area modified for pasture	Soil use	MAPBIOMAS.
NUrb	Percentage of total area modified for urbanization	Soil use	MAPBIOMAS.
NAct	Percentage of total area modified by human activities	Soil use	MAPBIOMAS.
Structure			
CHA	Number of community healthcare agents per inhabitant	Health	DATASUS
BHU	Number of basic health units per inhabitant	Health	DATASUS
Expenses	Health expenditure per inhabitant	Health	SIOPS
Outcome			
Coefficient100	If the municipality recorded less than five years of incidence below 100% of the coefficient by population size (1 (Best) = less than five years and 2 (Worst) = five years or more)	Health	DATASUS
Coeficient20	If the municipality recorded less than five years of incidence below 20% of the coefficient by population size (1 (Best) = less than five years and 2 (Worst) = five years or more)	Health	DATASUS

The evaluation model for this research contains 19 indicators, 14 of which relate to context, three to structure, and two to outcomes (Table 1). The indicators cover six areas: climate (three indicators), socioeconomic (three), sanitation (two), demographics (one), land use (four), and health (six). The health indicators correspond mainly to the two components of the Donabedian model evaluated in this study (structure and outcomes). Infant mortality was adopted as a context indicator, as this index can be used to assess the living conditions of the population^15^.

**Table 1:** Average values of the Context and Structure component indicators in relation to the outcome indicator classes.

Indicator	Coefficient100		Coeficient20	
	Class 1	Class 2	Class 1	Class 2
Total population	29,160,653	14,654,207	5,574,468	38,240,392
Incidence	464.44	1,199.38	230.05	872.21
HDI	0.73	0.75	0.72	0.75
Gini	0.46	0.46	0.46	0.46
Infant mortality	11.82	10.79	12.14	11.26
GDP *per capita*	26,738.83	30,332.25	22,090.17	30,387.45
% urban population	81.80	91.02	76.69	87.94
% population with water serv.	79.75	86.71	74.81	84.90
% population with sewage serv.	71.88	82.40	63.96	79.88
Maximum Temperature (°C)	27.11	28.59	26.17	28.15
Minimum temperature (°C)	15.88	17.07	15.09	16.73
Annual precipitation (mm^3^)	1,406.48	1,408.43	1,442.33	1,390.32
% Agricultural area	24.48	35.50	18.35	32.03
% Pasture area	21.87	18.07	21.84	20.35
% Urban area	5.21	5.47	3.39	6.17
% area modified by human activity	51.79	59.03	43.64	58.57
CHA per inhabitant.	1.47	1.31	1.46	1.40
BHU per inhabitant.	0.32	0.25	0.33	0.29
Expenditure per inhabitant	794.67	775.48	754.19	804.04

Source: Prepared by the authors.

Regarding the two outcome indicators, the guidelines from the Government of the State of São Paulo were used as references, which define an annual incidence coefficient according to the population size of the municipality^16^. This coefficient is used to classify municipalities according to the history of dengue transmission, a method that defines which means is used to assess the situation (histogram or control diagram).

### Data collection and storage

After defining the evaluation model, data were collected from public data sources (Chart). All data for the period from 2010 to 2019 were available except for climatic variables (2010 to 2018), HDI (2010 only), and Gini Index (2010 only).

The datasets were stored in their original forms on a cloud platform. The only modifications to data in this stage were related to climate data, which was only available in raster format*.* For this data, we extracted the mean temperature and precipitation values for each municipality, which were stored in comma-separated values (csv) format*.*

### Data Warehouse Creation

To perform the analyses, the data were integrated into *a* Data Warehouse (DW), a form of storage prepared for analytical operations. The operations are usually conducted using the extract-transform-load (ETL) model, in which the data is extracted from its original sources, transformed for necessary adjustments, such as standardization, and loaded into the DW for further analysis.

For this stage, Python scripts were developed and run in a cloud environment for each data set. The standard procedure for all scripts consisted of the following stages: collecting the dataset file from the cloud, performing the necessary modifications, and loading the data into the DW.

To integrate the data, the Brazilian Institute of Geography and Statistics (IBGE) code was used to identify each municipality since differences in the spelling of municipal names were identified between data sources. Even for this code, differences were found in the data sources, with some reporting the code in six digits and others in seven. These differences in data sources can often hinder data integration processes, making it necessary to develop automated procedures to transform them.

After the appropriate modifications, the data was loaded into a database hosted on the Google BigQuery, a cloud analytics platform that provides access to queries and machine learning algorithms.

### Data selection and preparation

To perform the analyses, automated queries were performed to extract the data stored in the DW created in the previous stage. With the collection, the following actions were performed:

- Standardization: as the variables are available in absolute values, operations were performed to standardize them in terms of the population;
- Calculation of incidence: the annual incidence of dengue cases was calculated for each municipality;
- Evaluation of the municipalities: for each year of the study period, the municipalities were evaluated according to two criteria: 1) whether the incidence rate was equal to or greater than 20% of the expected value for their population size^16^; and 2) whether the incidence rate was equal to or greater than 100% of the expected value for their population size^16^;
- Classification of municipalities: an indicator was created related to the total number of years for each criterion previously described. Each municipality was evaluated on both indicators according to two classes: 1) the class that indicates that the municipality recorded less than five years of incidence below the criterion; and 2) five or more years of incidence above the adopted criterion. The indicators generated were named *coefficient20* and *coefficient100* for criterion A and B, respectively.

The adoption of this classification criterion is based on the guidelines of the state of São Paulo to control dengue. Municipalities with no historical series are those whose incidence was 20% lower than expected according to population size, in at least five of the last ten years. Thus, these municipalities could perform better than those that have had an incidence higher than this threshold for more than five years. The second criterion (above or below 100%) was defined as an additional ranking metric, that is, to verify those municipalities that exceeded this expected incidence by at least five years.

### Data Analyses

Initially, statistical analyses were performed according to the two outcome indicators. The mean values of each context and structure indicator were calculated for each class to verify possible patterns.

Then, the K-Means algorithm was used to identify the groups according to the climatic conditions. The characteristic of this algorithm is the previous definition of the number of groups to be created^17^. According to the previous evaluation and with the aid of the Elbow method^18^, it was defined that there would be four groups. As climatic conditions greatly influence the proliferation of mosquitoes, we sought to create groups to make comparisons in municipalities with similar climatic conditions. An advantage of this method is that analyses can be performed on large volumes of data.

Once the groups had been created, statistical analyses were conducted for each one, estimating mean values for each indicator within the class of outcome indicators. The objective of this stage is to verify possible differences in context and structure in relation to the performance of municipalities in dengue control.

### Outcome Analyses

Finally, the outcomes were evaluated for the extraction of relevant information. In addition to the statistical analyses, spatial visualizations of the information were performed to identify possible patterns.

## OUTCOMES

### General Analysis

Table 1 shows the mean values for each indicator from 2010 to 2019 for the two classes of municipalities. With these data, some important differences between the two classes of municipalities can be observed.

For the *coefficient100* indicator, it is noted that the municipalities of class 2 (worst performance) hold, on average, better socioeconomic indicators (HDI, GDP, and GDP *per capita*). In addition, these municipalities present lower infant mortality rates and better sanitation conditions. Despite this, it is observed that they have more favorable conditions for the proliferation of mosquitoes, as they show a larger urban population and higher temperatures. Only these factors could explain the worse performance of class 2 municipalities in relation to this outcome indicator.

However, despite showing better socio-economic indicators and more favorable conditions, these municipalities presented lower numbers in the three indicators related to health structure *per capita*: number of community health agents, number of basic health units, and investments in health. The same pattern is observed in the analysis of the second outcome indicator (*coefficient20*), except for indicators related to the annual precipitation volume and investments in health *per capita*. In this case, the worst-performing municipalities (class 2) presented, on average, lower annual precipitation volume and higher investments in health *per capita*.

### Intergroup analysis

**Figure 1:**
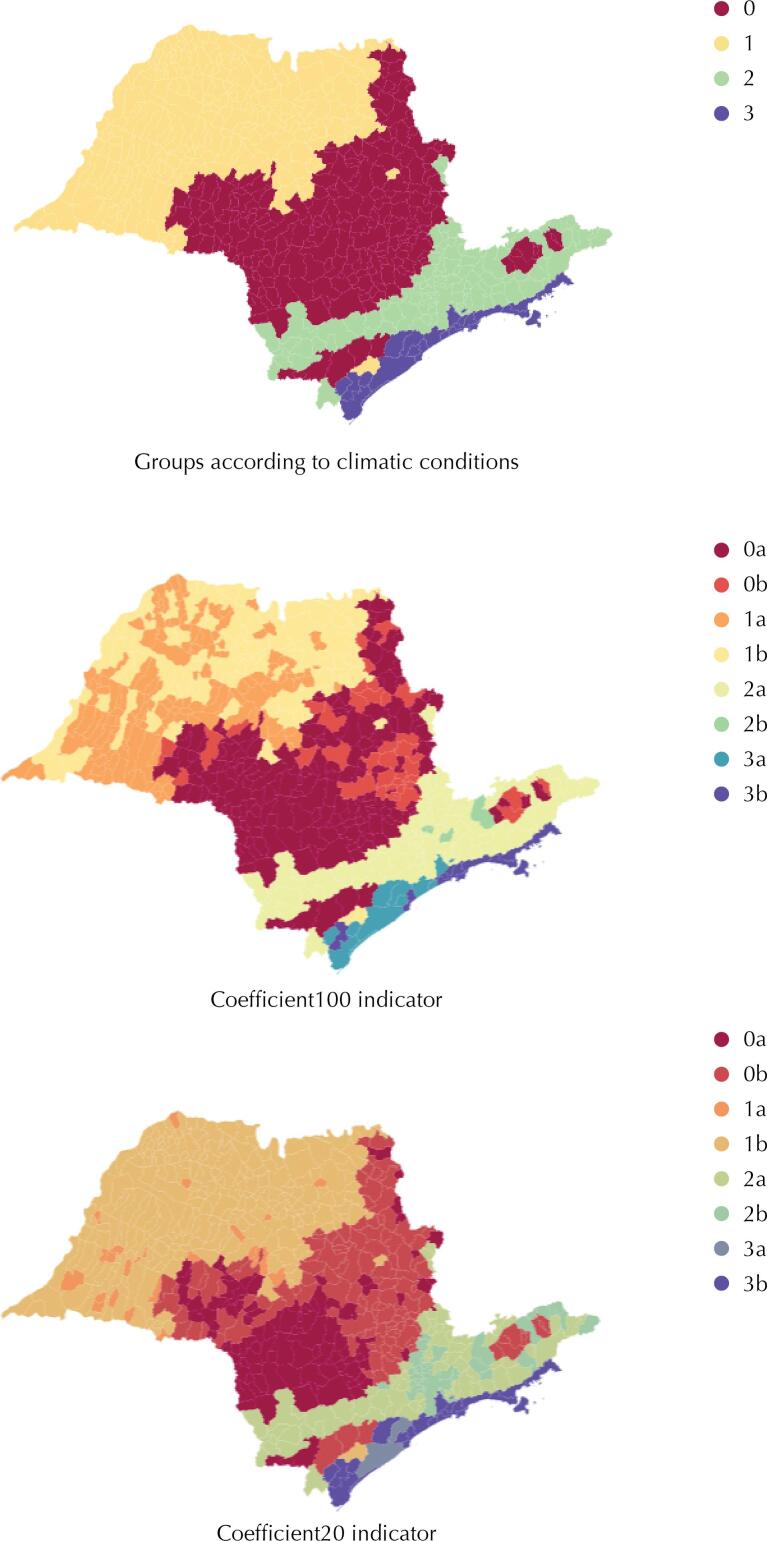
Municipalities grouped according to their climatic conditions and performance in terms of outcome indicators. Note: The number corresponds to the group and the letter to the class in terms of the performance of the indicator (a = best, b = worst).

Then, the municipalities were grouped according to their climatic conditions. According to Figure 1, it is possible to see a regional pattern between the groups, with Group 0 corresponding mainly to municipalities in the central region of the state; Group 1 to the North and West regions; Group 2 to the region that includes the Metropolitan Region of São Paulo and the Paraíba Valley; and Group 3 corresponding to the state’s coastline. The spatial visualization of the groups is a way to verify the validity of the clustering method employed. This pattern is similar to that found in another study conducted in the state of São Paulo, in which the relationship between the increase in temperature and the territorial expansion of the dengue vector over the years was verified^19^.

For each group, the mean values of each indicator were calculated, as shown in Table 2. In addition, the percentage of the total number of worst-performing municipalities in each group was calculated (with indicators *coefficient100* = 2 and *coefficient20* = 2). From this data, the two groups with the worst performance in both indicators (Clusters 1 and 3) perform differently in terms of the indicators. Regarding infant mortality, Group 1 obtained the best outcomes among the four groups, while the municipalities in Cluster 3 scored the highest. Regarding the Gini index, the opposite is true: the municipalities in Group 1 scored the lowest average value for this indicator, while the municipalities in Group 3 scored the highest values.

**Table 2:** Annual average values of each indicator by group.

Indicator	Group			
	0	1	2	3
Number of municipalities	236	274	111	24
Total population	11,092,927	6,115,605	23,502,689	3,103,638
Mean incidence	504.75	994.04	180.2	756.61
% of municipalities with the worst performance (Coefficient100 = 2)	16.95	44.16	3.60	50.00
% of municipalities with the worst performance (Coefficient20 = 2)	55.51	90.51	34.23	87.50
HDI	0.74	0.74	0.73	0.74
Gini	0.46	0.43	0.50	0.51
Infant mortality	11.60	10.96	12.41	13.45
GDP *per capita*	29,409.25	24,682.13	29,371.26	38,277.96
% urban population	84.91	85.22	79.88	89.04
% population with water serv.	83.19	82.56	76.43	80.58
% population with sewage serv.	77.88	80.13	59.23	54.78
Maximum Temperature (°C)	27.17	29.38	24.04	25.73
Temperature Minimum Temperature (°C)	15.72	17.37	14.02	17.78
Annual precipitation (mm^3^)	1,382.38	1,268.38	1,594.74	2,563.74
% Agricultural area	28.37	37.73	6.31	3.97
% Pasture area	18.44	27.02	14.87	1.14
% Urban area	4.46	1.45	15.76	8.93
% area modified by human activity	51.29	66.19	37.01	14.11
CHA per inhabitant.	1.21	1.74	1.07	1.50
BHU per inhabitant.	0.29	0.35	0.22	0.21
Expenditure per inhabitant	764.63	853.72	661.2	891.67

Source: Prepared by the authors.

While the HDI remained practically unchanged for the four groups, with only Group 2 showing a lower value, there was a great disparity in the GDP *per capita* values. The municipalities in Group 3 showed, on average, higher values, followed by groups 0 and 2 and, finally, *Cluster* 1. In terms of urban population, almost 90% of the population of Group 3 municipalities lived in urban areas compared to about 85% in Groups 0 and 1.

For water and sewerage, there are similar values for the indicator for water supply, whereas for sewage there are significant differences, especially for municipalities in groups 2 and 3, which mainly include municipalities in the Metropolitan Region of São Paulo, Vale do Paraíba, and Litoral Paulista.

Regarding climate variables, more favorable conditions (higher temperature and lower precipitation) are observed for the group of municipalities with the worst performance in the *coefficient20* indicator (Group 1). Despite these municipalities accounting for the smallest percentage of urban areas, the indicator for area modified by human activities recorded the highest values.

Regarding structural indicators, there were higher investments and higher numbers of community health workers per capita in the municipalities with the worst outcomes (groups 1 and 3).

### Intragroup analysis

Finally, the outcomes were evaluated internally in the groups, according to the classes of the outcome indicators. For this analysis, we estimated the mean values for the *coefficient20* (Table 3), a measure adopted in the guidelines of the São Paulo State Health Department to classify municipalities with or without a historical transmission series.

**Table 3:** Average values of the indicators in each group of municipalities regarding climatic conditions according to the classes of the *coefficient20*.

Group	0		1		2		3	
Class (2 = worst)	1	2	1	2	1	2	1	2
Number of municipalities	105	131	26	248	73	38	3	21
Incidence	225.83	728.31	531.74	1,042.50	118.89	297.99	467.8	797.86
HDI	0.72	0.75	0.73	0.74	0.72	0.76	0.72	0.74
Gini	0.46	0.47	0.41	0.44	0.49	0.51	0.48	0.51
Infant mortality	12.23	11.10	9.69	11.1	12.79	11.68	14.12	13.36
GDP *per capita* (x1000 Brazilian *reais*)	20.95	36.19	23.81	24.77	23.24	41.15	33.25	43.30
% urban population	79.08	89.59	78.31	85.95	72.02	94.34	85.00	89.62
% population with water serv.	78.82	86.70	77.46	83.09	68.12	92.47	70.48	82.02
% population with sewage serv.	70.72	83.62	74.72	80.70	51.36	75.68	43.41	56.41
Maximum Temperature (°C)	27.02	27.30	29.22	29.39	23.91	24.27	24.86	25.86
Minimum temperature (°C)	15.39	15.98	17.28	17.38	13.82	14.42	16.76	17.93
Annual precipitation (mm^3^)	1,353.30	1,405.70	1,264.10	1,268.80	1,605.00	1,575.10	2,144.40	2,395.10
% Agricultural area	22.57	33.03	32.77	38.26	7.73	3.59	4.43	3.91
% Pasture area	22.90	14.87	36.74	26.00	15.88	12.92	0.53	1.22
% Urban area	1.50	6.83	0.38	1.53	7.09	32.40	5.80	9.37
% area modified by human activity	47.00	54.73	70,01	65.79	30.77	48.98	10.73	14.59
CHA per inhabitant.	1.45	1.01	2.21	1.69	1.21	0.81	1.69	1.48
BHU per inhabitant.	0.33	0.25	0.48	0.33	0.27	0.14	0.28	0.21
Expenditure per inhabitant	762.93	765.99	1,043.7	833.8	646.7	689.05	555.2	939.75

Source: Prepared by the authors.

According to Table 3, in general, the worst-performing municipalities (Class 2) in the four groups follow the same pattern: better socioeconomic conditions and sanitation and larger urban population, but with lower values for the health structure component indicators.

For Group 2, it can be observed that the municipalities with the worst performance showed better socioeconomic conditions but a larger urban population and a significantly higher value for urbanization. Although investment in health is higher, these municipalities show a worse structure for the two primary care indicators. Regarding the municipalities in groups 0 and 3, the same differences identified for Group 2 are observed: better general conditions and greater investment in health but worse health structure.

A similar pattern is observed in Group 1, except for the indicator referring to investments in health. The worst-performing municipalities also showed lower average investment in health per inhabitant.

Finally, the outcomes for the two outcome indicators according to the performance classes can be visualized spatially in Figure 1. The first map shows the municipalities according to the four groups identified in the algorithm. In the following two maps, we have a classification (Best/Worst) for the two coefficients of outcomes defined in Chart 1. It can be observed, in general, that the worst performances are in the municipalities located further east of the state. By analyzing the other indicators spatially, we identified which range comprises the municipalities with the highest urban population rates.

## DISCUSSION

In this study, it was possible to aggregate contextual data for an evaluation of the Brazilian Dengue Control Program based on the Donabedian model for the 645 municipalities in the state of São Paulo. Although the program is the same for everyone, there are different municipal configurations, such as the number of professionals working against the vector, which does not always meet the standards of the national program. Although it is difficult to evaluate the quality of municipal work, these differences can influence the final quality of dengue control program.

Using the concepts of Planetary Health and Data Science techniques, data from various areas were integrated into a Data Warehouse to conduct the evaluations.

The context of Big Data, with greater availability of data sources, presents several opportunities to advance knowledge in healthcare. However, the extraction of useful information in Big Data requires extra methods to be efficient. The methodology adopted in this study allowed the evaluation of the dengue control program on a large scale, in addition to the integration of context data into a widespread model for health evaluations.

In the program’s evaluation, the deficiencies in health structure stood out in all three analyzed indicators for the municipalities with the best socioeconomic conditions, which did not necessarily imply better conditions in primary care and performance in terms of dengue cases. Although it cannot be said that these are the only reasons for the higher incidence of dengue, the differences observed in the indicators of these municipalities may suggest that economic development has not been accompanied by improvements in health conditions, especially considering that these places have a larger urban population.

Additionally, Table 1 suggests that, even with more favorable conditions for mosquito proliferation, such as higher temperatures and urban population, the municipalities with the worst performance in terms of outcome indicators showed a worse health structure than those with less favorable conditions, when the opposite would be expected. Considering only the outcome indicator *coefficient20*, the data in Table 1 show that about 38 million people live in areas more susceptible to mosquito proliferation and with worse health conditions in the two indicators related to primary care. Public management could consider this information to better target investments on the major needs of each location in the fight against dengue and other diseases related to the same factors.

As the Planetary Health studies suggest, unsustainable development comes at a cost to human health. Although some indicators may have improved, others have regressed, as can be seen in the indicators related to dengue.

A limitation of this study regards the observed data period. We used data up to 2019, which may be out of date, also considering the changes in health systems caused by the COVID-19 pandemic. In addition, due to delays in the Brazilian Census, some data from 2010, such as the HDI and the Gini Index, were used. Given the absence of the Census, which was to be conducted in 2020, population data were used based on estimates. Therefore, once updated data are available, new analyses can be conducted using the method presented in this study.
