# Supplementary material for: Big Data-Planetary Health approach for evaluating the Brazilian Dengue Control Program
Source: Rev Saude Publica. 2024 Apr 12;58:17. doi: 10.11606/s1518-8787.2024058005491 (PMC11037898; doi:10.11606/s1518-8787.2024058005491)
Supplement: Translation

# Translation: Abordagem Big Data-Saúde Planetária para avaliação do Programa de Combate à Dengue

**Authors:** Fernando Xavier, Gerson Laurindo Barbosa, Cristiano Corrêa de Azevedo Marques, Antonio Mauro Saraiva

**Affiliations:** I Brasil Universidade de São Paulo. Programa de Pós-Graduação em Engenharia Elétrica. São Paulo, SP, Brasil; II Brasil Secretaria de Estado da Saúde de São Paulo. Instituto Pasteur. Área Técnica de Doenças Vinculadas a Vetores e Hospedeiros Intermediários. São Paulo, SP, Brasil; III Brasil Universidade de São Paulo. Escola Politécnica. Departamento de Engenharia de Computação e Sistemas Digitais. São Paulo, SP, Brasil

**Keywords:** Epidemiologia, Dengue, Avaliação de Processos e Resultados em Cuidados de Saúde, Ciência de Dados

## Abstract

**OBJETIVO:**

Integrar os conceitos de Saúde Planetária e *Big Data* ao modelo de Donabedian, para avaliar o Programa de Combate à Dengue no estado de São Paulo.

**MÉTODOS:**

Foram adotados métodos de Ciência de Dados para integração e análise de dados relacionados à dengue, agregando o contexto aos componentes de estrutura e de resultado do modelo de Donabedian. Esses dados, considerando o período de 2010 a 2019, foram coletados de fontes como Datasus, Instituto Brasileiro de Geografia e Estatística (IBGE), *WorldClim* e MapBiomas, e integrados em um *Data Warehouse*. Para a identificação de grupos com contextos similares, foi utilizado o algoritmo K-means. Em seguida, foram realizadas análises estatísticas e visualizações espaciais dos grupos, considerando variáveis socioeconômicas, demográficas, solo, estrutura de saúde e casos de dengue.

**RESULTADOS:**

Com o uso das variáveis climáticas, o algoritmo K-means identificou quatro grupos de municípios com características similares. A comparação dos seus indicadores revelou certos padrões nos municípios com pior desempenho quanto aos resultados de casos de dengue. Embora tivessem melhores condições econômicas, eles tinham menor número médio de agentes comunitários e de unidades básicas de saúde por habitante. Dessa forma, as condições econômicas não refletiram em melhor estrutura de saúde nos três indicadores avaliados. Outra característica desses municípios é a urbanização. Os municípios de pior desempenho tinham maior taxa de população urbana e de modificações antrópicas relacionadas à urbanização.

**CONCLUSÕES:**

Por meio desta metodologia, foi possível identificar importantes deficiências nas condições para a execução do programa de combate à dengue no estado de São Paulo. A integração de diversas bases de dados e a utilização de métodos de Ciência de Dados permitiram a avaliação do programa em larga escala, considerando o contexto em que as ações são executadas. Dessa forma, a gestão pública pode utilizar as informações coletadas para planejar ações e investir de acordo com as deficiências de cada local.

## INTRODUÇÃO

O número de casos e óbitos por dengue vem aumentando a cada ano, superando marcas históricas. De acordo com o Boletim Epidemiológico do Ministério da Saúde referente à Semana Epidemiológica 51, em 2022^1^, foram relatados 992 óbitos confirmados por dengue, número superior a 2015 (986), quando tinha sido registrado o maior número até então^2^. Segundo o mesmo boletim, o estado de São Paulo foi a unidade da federação que apresentou o maior número de óbitos nesse ano (278), cerca de 28% do total registrado no país.

No Brasil, o controle vetorial do *Aedes* teve início em 1976, com ações esporádicas, e a partir de 2002, com a criação do Programa Nacional de Controle da Dengue (PNCD), foram desenvolvidas atividades rotineiras de combate ao vetor. Algumas dessas atividades, como pesquisa larvária amostral em domicílios, visitas em pontos estratégicos, visitas domiciliares objetivando orientação ao morador e eliminação de formas imaturas do vetor, atividades de bloqueio de transmissão quando necessário, entre outras, são desenvolvidas rotineiramente no estado de São Paulo, seguindo as diretrizes nacionais.

O vetor transmissor da dengue (o mosquito *Aedes aegypti*) encontra condições favoráveis para o seu desenvolvimento em locais com temperaturas mais altas e ambientes com alta urbanização, e, segundo projeções, esses lugares tendem a aumentar nos próximos anos^3^. Dada a forte influência das mudanças no ambiente para doenças como a dengue^4^, as mudanças climáticas podem contribuir para o aumento da proliferação de vetores. A urbanização sem planejamento adequado representa outra ameaça para diversas doenças infecciosas^5^ e, com a piora das condições de vida, o fluxo migratório pode aumentar ainda mais. Na última década, cerca de, em média, 24 milhões de pessoas por ano foram deslocadas de suas residências devido, principalmente, a eventos relacionados ao clima^6^.

A urbanização, embora possa ser vista como reflexo do desenvolvimento econômico, nem sempre ocorre de forma adequada^7^. A destruição dos ecossistemas e a ocupação desordenada dos espaços ocasiona diversos impactos nas condições de vida da população^7^. Enquanto alguns indicadores têm evolução, como a expectativa de vida, em outros observa-se uma piora, como a frequência de ocorrência de surtos de doenças^8^.

Essa complexa relação entre a saúde humana e as condições ambientais é tema de estudo da área chamada Saúde Planetária^9^, cujas pesquisas buscam avaliar o impacto do ambiente na saúde. As condições de saúde, que muitas vezes são um reflexo das ações antrópicas, estão relacionadas ao seu contexto. Os casos de dengue estão, entre outros fatores, relacionados às condições climáticas^10^ que, por sua vez, podem estar relacionadas às ações humanas^9^.

Dessa forma, a avaliação das condições de saúde pode estar relacionada ao contexto em que as ações são executadas. O modelo de avaliação de Donabedian^11^ considera que os resultados de saúde dependem de ações (processos) e condições dos serviços de saúde (estrutura). Assim, nesse modelo, as avaliações podem incluir análises dos três componentes que estão relacionados entre si: estrutura, processo e resultado.

A avaliação dos indicadores do componente estrutura permite analisar a situação da estrutura de saúde no local: a quantidade de profissionais, as unidades de atendimento, os investimentos, entre outros. Os indicadores de processo, por sua vez, são úteis para avaliar se as atividades estão sendo executadas conforme as normas, por exemplo. Esses dois componentes podem ser avaliados em conjunto com os indicadores de resultado, permitindo uma avaliação integrada das condições em que os resultados de saúde são produzidos.

No entanto, as condições e as ações necessárias para os resultados de saúde podem depender também do contexto em que estão inseridas^12^. Dessa forma, uma avaliação de saúde também poderia considerar variáveis relacionadas ao contexto.

Considerando a complexa relação entre os determinantes de saúde discutida na área de pesquisa da Saúde Planetária, métodos de avaliação em saúde, como o modelo de Donabedian, poderiam utilizar dados de diferentes dimensões, como economia, condições de vida e desenvolvimento urbano. Todavia, o aumento da complexidade no processo de avaliação de saúde pode tornar esse processo impeditivo por razões de custo e de tempo, especialmente no contexto de *Big Data*, em que há variadas fontes de dados gerados a velocidades cada vez maiores.

Nesse sentido, esta pesquisa tem como objetivo a adoção de técnicas de Ciência de Dados, como aplicação de algoritmos de aprendizado de máquina e análises estatísticas, para agregar a visão de contexto na avaliação do programa de combate à dengue com base no modelo de Donabedian. Dada a diversidade de variáveis relacionadas à dengue, o uso da Ciência de Dados pode trazer grandes benefícios para o tema da avaliação em saúde, como possibilitar que processos avaliativos possam ser executados em grande escala. Neste estudo, foram analisados dados de diversas variáveis dos 645 municípios do estado de São Paulo, entre 2010 e 2019.

## MÉTODOS

Pesquisas baseadas em Ciência de Dados consideram, além de técnicas computacionais e estatísticas, a inclusão dos especialistas do domínio^13^. Essa interação agrega a visão da área aos projetos de pesquisa, como a identificação das fontes de dados necessárias, a delimitação do problema e a avaliação dos resultados. Dessa forma, este estudo foi dividido em seis etapas, descritas a seguir, desde a definição do modelo de avaliação até a análise dos resultados.

### Definição do modelo de avaliação

Foram identificados os principais indicadores para a avaliação do programa de combate à dengue. Esse processo apoiou-se na literatura existente para agregar evidências formais para o uso dos indicadores^14^, assim como a validação, junto a especialistas, na matriz apresentada no Quadro 1. Para esta pesquisa, foram utilizados indicadores referentes aos componentes estrutura e resultado do modelo de Donabedian, além de aqueles relacionados ao contexto.

**Quadro:** Matriz de avaliação utilizada considerando os componentes Contexto, Estrutura e Resultado.

Indicador	Descrição	Tipo	Fonte
Contexto			
Tmax	Temperatura média máxima anual	Clima	WorldClim
Tmin	Temperatura média mínima anual	Clima	WorldClim
Prec	Volume de chuva anual	Clima	WorldClim
PIB	Produto interno produto *per capita*	Socioeconômico	IBGE
Gini	Índice Gini	Socioeconômico	Datasus
IDHM	Índice de desenvolvimento humano	Socioeconômico	IPEA
Mortalidade infantil	Taxa de mortalidade infantil por mil nascimentos	Saúde	Datasus
Água	Porcentagem da população atendida com abastecimento de água	Saneamento	SNIS
Esgoto	Porcentagem da população atendida com esgotamento sanitário	Saneamento	SNIS
População urbana	Porcentagem da população urbana	Demográfico	SNIS
NAgri	Porcentagem da área total modificada para atividades agrícolas	Uso do solo	MapBiomas
NPast	Porcentagem da área total modificada para pasto	Uso do solo	MapBiomas
NUrb	Porcentagem da área total modificada para urbanização	Uso do solo	MapBiomas
NAnt	Porcentagem da área total modificada por ações antrópicas	Uso do solo	MapBiomas
Estrutura			
ACS	Número de agentes comunitários de saúde por habitante	Saúde	Datasus
UBS	Número de unidades básicas de saúde por habitante	Saúde	Datasus
Despesas	Despesas em saúde por habitante	Saúde	SIOPS
Resultado			
Coeficiente100	Se o município teve menos de 5 anos com incidência abaixo dos 100% do coeficiente por porte populacional (1 (Melhor) = menos de cinco anos e 2 (Pior) = cinco anos ou mais)	Saúde	Datasus
Coeficiente20	Se o município teve menos de 5 anos com incidência abaixo dos 20% do coeficiente por porte populacional (1 (Melhor) = menos de cinco anos e 2 (Pior) = cinco anos ou mais)	Saúde	Datasus

O modelo de avaliação para esta pesquisa contém 19 indicadores, sendo 14 relacionados ao contexto, três relacionados à estrutura e dois relacionados aos resultados (Quadro 1). Os indicadores abrangem seis áreas, sendo elas: clima (três indicadores), socioeconomia (três), saneamento (dois), demografia (um), uso do solo (quatro) e saúde (seis). Os indicadores de saúde correspondem, principalmente, aos dois componentes do modelo de Donabedian avaliados neste estudo (estrutura e resultados). Adotou-se a mortalidade infantil como indicador de contexto, pois esse índice pode ser utilizado para avaliar as condições de vida da população^15^.

Em relação aos dois indicadores de resultado, foram utilizadas como referências as diretrizes do Governo do Estado de São Paulo, que definem um coeficiente de incidência anual de acordo com o porte populacional do município^16^. Esse coeficiente é utilizado para classificar os municípios quanto à frequência de série histórica de transmissão de dengue, método que define qual meio é adotado para avaliar a situação (histograma ou diagrama de controle).

### Coleta e armazenamento dos dados

Após a definição do modelo de avaliação, foi realizada a coleta dos dados junto às fontes de dados públicos (Quadro). Com exceção das variáveis climáticas (2010 a 2018), IDH (apenas 2010) e Índice Gini (apenas 2010), todos os dados para o período de 2010 a 2019 estavam disponíveis.

Os conjuntos de dados foram armazenados em suas formas originais em uma plataforma de nuvem. As únicas transformações realizadas nessa etapa foram relacionadas aos dados climáticos, que estavam disponíveis apenas no formato *raster*. Para esses dados, extraímos os valores médios de temperatura e chuva para cada município, que foram armazenados em arquivos no formato *comma-separated values* (csv).

### Criação do Data Warehouse

Para a execução das análises, os dados foram integrados em *Data Warehouse* (DW), uma forma de armazenamento preparada para operações analíticas. As operações são realizadas, normalmente, no modelo *extract-transform-load* (ETL), em que os dados são extraídos de suas fontes originais, transformados para ajustes necessários, como padronização, e carregados no DW para análises posteriores.

Para essa etapa, foram desenvolvidos scripts em *Python*, que tiveram execução em um ambiente em nuvem, para cada conjunto de dados. O procedimento padrão para todos os scripts era composto dos seguintes passos: coleta do arquivo do conjunto de dados na nuvem, execução das transformações necessárias e carregamento dos dados no DW.

Para a integração dos dados, foi utilizado o código IBGE como identificador único do município, visto que foram identificadas diferenças na grafia dos nomes dos municípios entre as fontes de dados. Mesmo para esse código foram encontradas diferenças nas fontes de dados, com algumas informando o código em seis dígitos e outras em sete. Essas diferenças nas fontes de dados podem, muitas vezes, dificultar os processos de integração de dados, fazendo com que seja necessário o desenvolvimento de procedimentos automatizados para transformá-los.

Após as devidas transformações, os dados foram carregados em um banco hospedado na ferramenta *Google BigQuery*, plataforma analítica em nuvem que provê acesso a consultas e execução de algoritmos de aprendizado de máquina.

### Seleção e preparação dos dados

Para a execução das análises, foram realizadas consultas automatizadas para a extração dos dados armazenados no DW criado no passo anterior. Com a coleta, as seguintes atividades foram realizadas:

- Padronização: como as variáveis são disponibilizadas em valores absolutos, foram executadas operações para sua padronização quanto à população;
- Cálculo da incidência: foi realizado o cálculo da incidência anual dos casos de dengue para cada município;
- Avaliação dos municípios: para cada ano do período do estudo, os municípios foram avaliados em dois critérios: 1) se a taxa de incidência foi igual ou superior a 20% do valor esperado para seu porte populacional^16^; e 2) se a taxa de incidência foi igual ou superior a 100% do valor esperado para seu porte populacional^16^;
- Classificação dos municípios: foi criado um indicador relacionado ao total de anos para cada critério definido anteriormente. Cada município foi avaliado nos dois indicadores, de acordo com duas classes, sendo: 1) a classe que indica que o município teve menos de cinco anos com incidência abaixo do critério; e 2) com cinco ou mais anos com incidência acima do critério adotado. Os indicadores gerados foram nomeados de *coeficiente20*, para o critério A, e *coeficiente100*, para o critério B.

A adoção desse critério de classificação é baseada nas diretrizes do estado de São Paulo para combate à dengue. São considerados municípios sem série histórica aqueles cuja incidência foi 20% menor que o esperado de acordo com o porte populacional, em pelo menos cinco dos últimos dez anos. Assim, esses municípios poderiam ter melhor desempenho em relação aos que tiveram incidência maior do que esse limite em mais de cinco anos. O segundo critério (acima ou abaixo de 100%) foi definido como uma métrica adicional de classificação, ou seja, para verificar aqueles municípios que ultrapassaram essa incidência esperada em pelo menos cinco anos.

### Execução das análises

Inicialmente, foram realizadas análises estatísticas em função dos dois indicadores de resultados. Foram calculados os valores médios de cada indicador de contexto e de estrutura para cada classe, de forma a verificar possíveis padrões.

Em seguida, foi executado o algoritmo K-means para identificar os grupos, de acordo com as condições climáticas. Esse algoritmo tem como característica a definição prévia do número de grupos a serem criados^17^. De acordo com a avaliação prévia e com o auxílio do método Elbow^18^, definiu-se que seriam quatro grupos. Como as condições climáticas têm grande influência na proliferação dos mosquitos, buscou-se a criação de grupos para a realização das comparações em municípios com condições climáticas semelhantes. Uma vantagem desse método é que as análises podem ser realizadas em grandes volumes de dados.

Após a criação dos grupos, foram realizadas análises estatísticas para cada um, com o cálculo dos valores médios de cada indicador dentro da classe referente aos indicadores de resultados. O objetivo dessa etapa foi verificar possíveis diferenças quanto ao contexto e à estrutura, em relação ao desempenho dos municípios no combate à dengue.

### Análise dos resultados

Por fim, os resultados foram avaliados para a extração de informação relevante. Além das análises estatísticas, foram realizadas visualizações espaciais das informações para uma possível identificação de padrões.

## RESULTADOS

### Análise geral

Na Tabela 1 são apresentados os valores médios no período entre 2010 e 2019 de cada indicador para as duas classes de municípios. Com esses dados, podem ser observadas algumas diferenças importantes entre as duas classes de municípios.

**Tabela 1:** Valores médios dos indicadores dos componentes Contexto e Estrutura em relação às classes dos indicadores de resultado.

Indicador	Coeficiente100		Coeficiente20	
	Classe 1	Classe 2	Classe 1	Classe 2
População total	29.160.653	14.654.207	5.574.468	38.240.392
Incidência	464,44	1199,38	230,05	872,21
IDH	0,73	0,75	0,72	0,75
Gini	0,46	0,46	0,46	0,46
Mortalidade infantil	11,82	10,79	12,14	11,26
PIB *per capita*	26.738,83	30.332,25	22.090,17	30.387,45
% população urbana	81,80	91,02	76,69	87,94
% pop com abastec. Água	79,75	86,71	74,81	84,90
% pop com esgotamento sanitário	71,88	82,40	63,96	79,88
Temp. máx. (º C)	27,11	28,59	26,17	28,15
Temp. mín. (º C)	15,88	17,07	15,09	16,73
Chuva anual (mm3)	1.406,48	1.408,43	1.442,33	1.390,32
% Área agri.	24,48	35,50	18,35	32,03
% Área past.	21,87	18,07	21,84	20,35
% Área urb.	5,21	5,47	3,39	6,17
% área com modificações antrópicas	51,79	59,03	43,64	58,57
ACS por hab.	1,47	1,31	1,46	1,40
UBS por hab.	0,32	0,25	0,33	0,29
Despesa por hab.	794,67	775,48	754,19	804,04

Fonte: Elaborado pelos autores.

Para o indicador *coeficiente100*, nota-se que os municípios da classe 2 (pior desempenho) têm, em média, melhores indicadores socioeconômicos (IDH, PIB e PIB *per capita*). Além disso, esses municípios têm menores taxas de mortalidade infantil e melhores condições de saneamento. Apesar disso, observa-se que dispõem de condições mais favoráveis para a proliferação de mosquitos, por terem maior população urbana e temperaturas mais altas. Apenas esses fatores poderiam explicar o pior desempenho dos municípios da classe 2 em relação a esse indicador de resultados.

No entanto, mesmo com melhores indicadores socioeconômicos e mais condições favoráveis, esses municípios apresentam números inferiores nos três indicadores relacionados à estrutura em saúde *per capita*: número de agentes comunitários de saúde, número de unidades básicas de saúde e investimentos em saúde. O mesmo padrão é observado na análise do segundo indicador de resultado (*coeficiente20*), com exceção dos indicadores relacionados ao volume anual de chuvas e aos investimentos em saúde *per capita*. Nesse caso, os municípios de pior desempenho (classe 2) tiveram, em média, menor volume de chuva anual e maiores investimentos em saúde por habitante *per capita*.

### Análise intergrupos

Em seguida, os municípios foram agrupados de acordo com suas condições climáticas. De acordo com a Figura, é possível observar um padrão regional entre os grupos, com o Grupo 0 correspondendo principalmente aos municípios da região central do estado; o Grupo 1 às regiões Norte e Oeste; o Grupo 2 à faixa que inclui a Região Metropolitana de São Paulo e o Vale do Paraíba; e o Grupo 3 correspondendo ao litoral do estado. A visualização espacial dos grupos é uma forma de verificar a validade do método de clusterização empregado. Esse padrão é semelhante ao encontrado em outro trabalho realizado no estado de São Paulo, em que foi verificada a relação do aumento de temperatura à expansão territorial do vetor da dengue ao longo dos anos^19^.

**Figura:** Municípios agrupados de acordo com suas condições climáticas e desempenho quanto aos indicadores de resultado.

Nota: O número corresponde ao grupo e a letra à classe quanto ao desempenho do indicador (a = melhor, b = pior).

Para cada grupo, foram calculados os valores médios de cada indicador, conforme Tabela 2. Além disso, foi calculada a porcentagem do número total de municípios com pior desempenho em cada grupo (com indicadores *coeficiente100* = 2 e *coeficiente20* = 2). Por meio desses dados, é possível observar que os dois grupos com piores desempenho nos dois indicadores (*clusters* 1 e 3) apresentam desempenho diverso quanto aos indicadores. Em relação à mortalidade infantil, o Grupo 1 obteve melhores resultados entre os quatro grupos, enquanto os municípios do *Cluster* 3 tiveram o maior valor. Em relação ao índice Gini, ocorre o inverso: os municípios do Grupo 1 tiveram o valor médio mais baixo para esse indicador, enquanto os municípios do Grupo 3 tiveram os valores mais altos.

**Tabela 2:** Valores médios anuais de cada indicador por grupo.

Variáveis	Grupo			
	0	1	2	3
Número de municípios	236	274	111	24
População total	11.092.927	6.115.605	23.502.689	3.103.638
Incidência média	504,75	994,04	180,20	756,61
% de municípios com pior desempenho (Coeficiente100 = 2)	16,95	44,16	3,60	50,00
% de municípios com pior desempenho (Coeficiente20 = 2)	55,51	90,51	34,23	87,50
IDH	0,74	0,74	0,73	0,74
Gini	0,46	0,43	0,50	0,51
Mortalidade infantil	11,6	10,96	12,41	13,45
PIB *per capita*	29.409,25	24.682,13	29.371,26	38.277,96
% população urbana	84,91	85,22	79,88	89,04
% pop com abastec. água	83,19	82,56	76,43	80,58
% pop com esgotamento sanitário	77,88	80,13	59,23	54,78
Temp. máx. (º C)	27,17	29,38	24,04	25,73
Temp. Mín. (º C)	15,72	17,37	14,02	17,78
Chuva anual (mm3)	1.382,38	1.268,38	1.594,74	2.563,74
% área agri.	28,37	37,73	6,31	3,97
% área past.	18,44	27,02	14,87	1,14
% área urb.	4,46	1,45	15,76	8,93
% área com modificações antrópicas	51,29	66,19	37,01	14,11
ACS por hab.	1,21	1,74	1,07	1,50
UBS por hab.	0,29	0,35	0,22	0,21
Despesa por hab.	764,63	853,72	661,2	891,67

Fonte: Elaborado pelos autores.

Enquanto o IDH manteve-se praticamente padrão para os quatro grupos, com valor abaixo apenas para o Grupo 2, observa-se uma grande disparidade nos valores do PIB *per capita*. Os municípios do Grupo 3 têm, em média, valores mais altos, seguidos pelos grupos 0 e 2 e, por fim, o *Cluster* 1. Em relação à população urbana, os municípios do Grupo 3 têm quase 90% dos seus habitantes vivendo em áreas urbanas, enquanto os municípios dos Grupo 0 e 1 têm em torno de 85%.

Para o saneamento, observa-se valores similares para o indicador referente ao abastecimento de água, enquanto para o esgotamento sanitário há sensíveis diferenças, especialmente para os municípios dos grupos 2 e 3, que compreendem principalmente os municípios da Região Metropolitana de São Paulo, do Vale do Paraíba e do Litoral Paulista.

Em relação às variáveis climáticas, observa-se condições mais favoráveis (maior temperatura e menor volume de chuva) para o grupo de municípios com pior desempenho no indicador *coeficiente20* (Grupo 1). Embora esses municípios tenham a menor porcentagem de área urbana, observa-se o maior valor no indicador referente às modificações antrópicas no ambiente.

No que concerne aos indicadores de estrutura, observa-se maiores investimentos e número de agentes comunitários de saúde *per capita* nos municípios com piores resultados (grupos 1 e 3).

### Análise intragrupos

Por fim, os resultados foram avaliados internamente nos grupos, de acordo com as classes dos indicadores de resultado. Para essa análise, calculou-se os valores médios para o indicador *coeficiente20* (Tabela 3), medida adotada nas diretrizes da Secretaria de Estado da Saúde de São Paulo para classificar os municípios com/sem série histórica de transmissão.

**Tabela 3:** Valores médios dos indicadores em cada grupo de municípios quanto às condições climáticas de acordo com as classes do indicador *coeficiente20*.

Grupo	0		1		2		3	
Classe (2 = pior)	1	2	1	2	1	2	1	2
Número de municípios	105	131	26	248	73	38	3	21
Incidência	225,83	728,31	531,74	1.042,50	118,89	297,99	467,8	797,86
IDH	0,72	0,75	0,73	0,74	0,72	0,76	0,72	0,74
Gini	0,46	0,47	0,41	0,44	0,49	0,51	0,48	0,51
Mortalidade infantil	12,23	11,10	9,69	11,10	12,79	11,68	14,12	13,36
PIB *per capita* (x 1000 reais)	20,95	36,19	23,81	24,77	23,24	41,15	33,25	43,30
% população urbana	79,08	89,59	78,31	85,95	72,02	94,34	85	89,62
% pop com abastec. água	78,82	86,70	77,46	83,09	68,12	92,47	70,48	82,02
% pop com esgotamento sanitário	70,72	83,62	74,72	80,70	51,36	75,68	43,41	56,41
Temp. máx. (º C)	27,02	27,30	29,22	29,39	23,91	24,27	24,86	25,86
Temp. mín. (º C)	15,39	15,98	17,28	17,38	13,82	14,42	16,76	17,93
Chuva anual (mm3)	1.353,30	1.405,70	1.264,10	1.268,80	1.605,00	1.575,10	2.144,00	2.395,10
% área agri.	22,57	33,03	32,77	38,26	7,73	3,59	4,43	3,91
% área past.	22,9	14,87	36,74	26,00	15,88	12,92	0,53	1,22
% área urb.	1,50	6,83	0,38	1,53	7,09	32,40	5,80	9,37
% área com modificações antrópicas	47,00	54,73	70,01	65,79	30,77	48,98	10,73	14,59
ACS por hab.	1,45	1,01	2,21	1,69	1,21	0,81	1,66	1,48
UBS por hab.	0,33	0,25	0,48	0,33	0,27	0,14	0,28	0,21
Despesa por hab.	762,93	765,99	1043,7	833,8	646,7	689.05	555,2	939,75

Fonte: Elaborado pelos autores.

De acordo com os dados da Tabela 3, pode-se observar que, em geral, os municípios com pior desempenho (classe 2) nos quatro grupos seguem o mesmo padrão: melhores condições socioeconômicas e saneamento, maior população urbana, mas com menores valores para os indicadores do componente de estrutura em saúde.

Para o Grupo 2, pode-se observar que os municípios com piores desempenho têm melhores condições socioeconômicas, mas apresentam maior população urbana e valor sensivelmente maior para a urbanização. Apesar do investimento em saúde ser superior, esses municípios têm pior estrutura para os dois indicadores da atenção básica. Em relação aos municípios dos grupos 0 e 3, são observadas as mesmas diferenças identificadas para o Grupo 2: melhores condições gerais e maior investimento em saúde, mas pior estrutura em saúde.

Um padrão parecido é observado em relação ao Grupo 1, com exceção do indicador referente aos investimentos em saúde. Os municípios de pior desempenho têm também menor investimento médio em saúde por habitante.

Por fim, os resultados para os dois indicadores de resultados de acordo com as classes de desempenho são visualizados espacialmente na Figura. No primeiro mapa são apresentados os municípios de acordo com os quatro grupos identificados no algoritmo. Nos dois mapas seguintes, temos uma classificação (Melhor/Pior) para os dois coeficientes de resultados definidos no Quadro 1. Pode-se observar, em geral, que os piores desempenhos estão nos municípios localizados mais a leste do estado. Por meio da análise espacial dos demais indicadores, identificou-se qual faixa compreende os municípios com maior taxa de população urbana.

## DISCUSSÃO

Neste estudo, foi possível agregar dados de contexto para uma avaliação do programa de combate à dengue baseada no modelo de Donabedian para os 645 municípios do estado de São Paulo. Apesar do programa ser o mesmo para todos, há diferentes configurações municipais, como a quantidade de profissionais de combate ao vetor que nem sempre atende às normas do programa nacional. Embora seja difícil avaliar a qualidade do trabalho municipal, essas diferenças podem influenciar na qualidade final do controle.

Utilizando os conceitos de Saúde Planetária e as técnicas de Ciência de Dados, integrou-se dados de diversas áreas em um *Data Warehouse* para conduzir as avaliações.

O contexto de *Big Data*, com maior disponibilidade de fontes de dados, apresenta diversas oportunidades para avançar no conhecimento na área da saúde. No entanto, a extração de informação útil em *Big Data* demanda métodos para realizar essas atividades de forma eficiente. Nesse sentido, a metodologia adotada neste trabalho possibilitou a avaliação do programa de combate à dengue em larga escala, além da integração de dados de contexto a um modelo bastante difundido para avaliações em saúde.

Na avaliação do programa, chama a atenção as deficiências de estrutura em saúde nos três indicadores analisados para os municípios com melhores condições socioeconômicas, que não implicaram necessariamente melhores condições na atenção básica e no desempenho quanto aos casos de dengue. Embora não se possa afirmar que essas são as únicas razões para a maior incidência de dengue, as diferenças observadas nos indicadores desses municípios podem sugerir que o desenvolvimento econômico não foi acompanhado por melhoria nas condições de saúde, especialmente considerando que esses locais têm maior população urbana.

Além disso, os dados da Tabela 1 sugerem que, mesmo com condições mais favoráveis para a proliferação de mosquitos, como maior temperatura e população urbana, os municípios com pior desempenho para os indicadores de resultado têm pior estrutura em saúde do que os locais com condições menos favoráveis, quando seria esperado o contrário. Considerando apenas o indicador de resultado *coeficiente20*, os dados da Tabela 1 mostram que cerca de 38 milhões de pessoas vivem em áreas mais suscetíveis à proliferação de mosquitos e com piores condições de saúde nos dois indicadores relacionados à atenção básica. A gestão pública poderia levar em conta essas informações para melhor direcionar os investimentos referentes às principais necessidades de cada local, em relação ao combate à dengue e a outras doenças relacionadas aos mesmos fatores.

Como sugerem os estudos sobre Saúde Planetária, o desenvolvimento de forma não sustentável tem um custo para a saúde humana. Embora possa haver evolução em alguns indicadores, há retrocesso em outros, como é possível observar nos indicadores relacionados à dengue.

Uma limitação deste estudo está relacionada ao período de dados observado. Utilizou-se dados até 2019, que podem estar defasados, considerando também as modificações nos sistemas de saúde, ocasionadas pela pandemia da covid-19. Além disso, devido aos atrasos na realização do Censo, utilizou-se alguns dados de 2010, como o IDH e o Índice Gini. Além disso, dada a ausência do Censo, que seria realizado em 2020, os dados populacionais foram usados com base em estimativas. Dessa forma, uma vez que os dados mais atuais estejam disponíveis, novas análises poderão ser conduzidas, utilizando o método apresentado neste estudo.
